# Activation of human B cells negatively regulates TGF-β1 production

**DOI:** 10.1186/s12974-017-0798-5

**Published:** 2017-01-19

**Authors:** Nicolas Molnarfi, Kristbjörg Bjarnadóttir, Mahdia Benkhoucha, Catherine Juillard, Patrice H. Lalive

**Affiliations:** 1 Department of Pathology and Immunology, Faculty of Medicine, University of Geneva, Geneva, Switzerland; 2 Unit of Neuroimmunology and Multiple Sclerosis, Division of Neurology, Department of Clinical Neurosciences, Geneva University Hospital, Geneva, Switzerland; 3 Department of Genetic and Laboratory Medicine, Geneva University Hospital, Geneva, Switzerland

**Keywords:** B cells, TGF-β1, Regulation, Human, Multiple sclerosis

## Abstract

**Background:**

Accumulating evidence indicate that B cells can exhibit pro- or anti-inflammatory activities. Similar to interleukin (IL)-10–competent B cells, we recently showed that transforming growth factor (TGF)-β1-producing regulatory B cells limit the induction of autoimmune neuroinflammation in mice, making them potentially important in maintaining peripheral immune tolerance in central nervous system inflammatory demyelinating disorders such as multiple sclerosis.

**Methods:**

In this study, we compared B cell production of TGF-β1 and IL-10, the two most studied regulatory cytokines, and the pro-inflammatory B cell-derived IL-6 and tumor necrosis factor cytokines under basal conditions and following polyclonal stimulation with dual B cell receptor (BCR) cross-linking and Toll-like receptor (TLR)9 engagement.

**Results:**

We showed that resting TGF-β1–producing B cells fall within both the naïve (CD27^−^) and memory (CD27^+^) B cell compartments. We found no spontaneous B cell-derived IL-10, IL-6 or tumor necrosis factor (TNF) production. Human B cell activation with anti-Ig antibodies plus CPG-B leads to only modest IL-10 production by memory CD19^+^CD27^+^ B cells while expression levels of IL-6 and TNF by both naive and memory B cells were strongly induced. Remarkably, stimulated B cells showed significantly reduced capacity to produce TGF-β1.

**Conclusions:**

These findings indicate that B cell activation may facilitate the development of excessive immune responses and autoimmunity by restricting B cell-derived TGF-β1 production by resting B cells and favoring in turns the proinflammatory actions of activated cytokine-producing B cells.

**Electronic supplementary material:**

The online version of this article (doi:10.1186/s12974-017-0798-5) contains supplementary material, which is available to authorized users.

## Background

Recent years have seen a significant increase in the interest of the roles of B cells in autoimmune diseases such as multiple sclerosis (MS), not only as precursors of antibody-producing plasma cells [1, 2], but also as key regulators of T cell activation and differentiation through their antigen presentation function [3, 4] and cytokine production [3, 5, 6]. To date, the strongest evidence for B cells playing a crucial role in the immune pathology of MS arises from clinical trials uncovering the effect and potency of anti-CD20 B cell-depleting therapies (BCDT) [7–10]. In addition, a growing body of experimental studies suggests that activation of antigen-driven B cells can lead to central nervous system (CNS) autoimmune reactions [11, 12] and that B cells in MS may be inherently polarized towards a functional proinflammatory phenotype [6, 13–16]. Remarkably, some recent work has reported evidences of increased proinflammatory cytokine responses of B cells from MS patients upon non-CNS specific (i.e., CpG DNA) stimulation [17–19], suggesting that abnormal B cell effector cytokine responses in MS patients are not restricted to specific autoreactive B cells.

Recent research indicates that the activation status dictates the diverse roles a respective B cell may play in MS pathogenesis [13]. Activated B cells, in particular memory B cells, are more pathogenic than naïve ones. While memory B cells are more easily activated to produce proinflammatory cytokines such as tumor necrosis factor (TNF), interleukin (IL)-6, and granulocyte macrophage-colony stimulating factor (GM-CSF), naïve B cells preferentially secrete the regulatory cytokine IL-10 [6, 17]. Although the current data support the conceptual idea of a prominent involvement of activated proinflammatory B cells in MS pathogenesis, accumulating experimental and clinical findings indicate that not all B cells play a pathogenic role in MS [20]. Defects in regulatory B cell functions have been documented in MS patients [13, 17, 21–23], suggesting that a disrupted balance between proinflammatory and suppressive B cell properties may be particularly relevant to the regulation of CNS autoimmunity. While exacerbation of MS activity as a result of anti-CD20-mediated B cell depletion has not yet been documented, increased proinflammatory monocytic activity following B cell depletion has been reported in experimental autoimmune encephalomyelitis (EAE) [24], a model for MS, and more recently in some anti-CD20 monoclonal antibodies-treated MS patients [25]. Evidence that BCDT also depletes potential protective B cell responses comes from clinical reports showing that infusion of the monoclonal anti-CD20 antibody Rituximab for autoimmunity has led to severe exacerbation of colitis and the spontaneous onset of colitis and psoriasis readily after the initiation of treatment [26–29]. Management of non-Hodgkin’s lymphoma with Rituximab has also been associated with the onset of autoimmunity [30]. Interestingly, in MS, treatment with Atacicept, a decoy receptor for the B cell growth factors B-cell activating factor (BAFF) and a proliferation-inducing ligand (APRIL), was found to exacerbate MS, possibly by altering regulatory B cell functions [31]. These cautionary data emphasize that pan depletion of B cells can be deleterious in some situations, and therefore supports further development of a therapeutic option for treating MS patients that spares regulatory B cell functions [32].

One of the first studies illustrating a decreased regulatory B cell function in patients with an autoimmune condition was performed in the context of MS [17]. Decreased IL-10 production was seen upon B cell activation via CD40, or B cell receptor (BCR) in conjunction with CD40 [17], or Toll-like receptor (TLR)9 [21, 23], indicating a general alteration of B cell functions in MS rather than a defect in certain activation signaling pathways. Similar to IL-10-producing B cells [33–35], transforming growth factor (TGF)-β1-producing regulatory B cells have recently been shown by our group to restrain the initiation phase of experimental autoimmune neuroinflammation [36], making them potentially important in maintaining peripheral immune tolerance in organ-specific autoimmune disease such as MS. Consistent with this premise, recent evidence indicates that B cell subpopulations expressing TGF-β can control regulatory T cell induction, immune tolerance promotion, and/or innate and adaptive immune response suppression [37–50]. With regards to the importance of TGF-β1-producing B cells in the regulation of CNS autoimmunity, evaluating whether B cell activation governs TGF-β1 expression by human B cells may provide a better understanding of the contribution and mechanism of regulatory B cell functions in autoimmune manifestations.

Here, we measured B cell production of TGF-β1 and IL-10, the two most studied regulatory cytokines, and the proinflammatory B cell-derived IL-6 and TNF cytokines under basal conditions and following polyclonal stimulation with dual BCR cross-linking and TLR9 engagement. Spontaneous TGF-β1 production by resting B cells was observed in both the naïve (CD27^−^) and memory (CD27^+^) B-cell compartments. In contrast, we found that IL-10 production was negligible in B cells under basal culture conditions. Likewise, unstimulated B cells did not produce the proinflammatory cytokines IL-6 and TNF. Remarkably, B cells stimulated with combined anti-Ig antibodies + CpG-B showed decreased TGF-β1 production capacity. We also noted that once activated, a fraction of memory, but not naïve, B cells expressed low, but significant, IL-10 levels. Finally, B cell stimulation strongly enhanced production of IL-6 and TNF by B cells. These findings indicate that B cell activation may contribute to immunological abnormalities seen in autoimmune disorders such as MS by restricting the regulatory functions of resting TGF-β1-producing B cells and in turn favoring the proinflammatory effects of activated B cells.

## Methods

### Standard protocol approvals

Peripheral blood B cells were isolated from buffy coats of blood from healthy volunteers (five male and one female, median age 55 years, range 29–67 years) provided by the Geneva Hospital Blood Transfusion Center. In accordance with the ethical committee of the Geneva Hospital, the blood bank obtained informed consent from the donors that a part of their blood would be used for research purposes.

### Cell preparation and B cell purification

Highly purified CD19^+^ B cells (<90% purities) were isolated from the mononuclear fraction using negative selection microbeads (Human B cell Enrichment Kit; EasySep 19054, STEMCELL Technologies).

### Reagents

AffiniPure F(ab')2 Fragment Goat Anti-Human IgA + IgG + IgM (H + L) (anti-Ig) was from Jackson ImmunoResearch. ODN 2006 (ODN 7909) Class B CpG oligonucleotide; a human TLR9 ligand, was purchased from Invivogen. X-Vivo 15 medium for culturing B cells was from Lonza.

### Staining and immunofluorescence analysis

Cells (10^6^ cells/mL) were suspended in complete medium (X-Vivo 15 medium containing 200 mg/mL penicillin, 200 U/mL streptomycin, 4 mM L-Glutamine and 1 mM Sodium Pyruvate, all from Gibco) in the presence of CpG-B 2006 (10 μg/mL, Tib Molbiol) and anti-Ig (10 μg/mL) in 24-well flat-bottom plates for 24 h, at 37 °C. During the last 5 h, cells were additionally stimulated with 50 ng/mL of phorbol myristate acetate (PMA), 1 nM ionomycin (Sigma-Aldrich), and 2 mM monensin (all from eBioscience). Viability was assessed using a UV Live/Dead Fixable dead cell staining kit (Invitrogen). Nonspecific antibody (Ab) interactions of Fc receptors (FcRs) on cells were limited using TruStain FcX reagent (BioLegend), as per the manufacturer’s instructions. Cells were stained with antibodies to the following cell surface markers: CD19–FITC, CD19–APC, CD27–FITC, and CD27–PE (all from BioLegend), isotype-matched controls were from Biolegend or BD Pharmingen. After washing, cells were fixed and permeabilized using a Fix and Perm kit (Invitrogen) and stained by intracellular staining (ICS) with IL-10–PE-Cy7, IL–6-PE, TNF–PerCP-Cy5.5 (all from BioLegend), and anti-human TGF-β1–APC mAb (R&D). Isotype controls were from BioLegend and R&D. After the cells were stained, they were washed twice and acquired within 1 h. For cell-surface LAP staining, B cells were stimulated as described above without the addition of monensin and stained after non-specific binding (TruStain FcX reagent) with anti-human LAP–PE-Cy7 (TGF-β1) (BioLegend) and appropriate PE-Cy7 mouse IgG1 κ isotype-matched control antibody (BD Biosciences). Samples were run through a FACS Cyan flow cytometer (Becton Dickinson) with standard equipment. Normalized median fluorescence intensity (MFI) was calculated by subtracting the MFI of isotype control antibody-stained cells from the MFI of specific antibody-stained cells. Gating strategy for B cell phenotyping is shown in Additional file 1: Figure S1A.

### Analysis of cytokine production

For ELISA assays, B cells (4 × 10^5^) were cultured in 250 μL of serum-free complete X-Vivo 15 medium with CpG-B 2006 (10 μg/mL) + anti-Ig (10 μg/mL) in 96-well flat-bottom plates. At 24 h, cell-free supernatants were analyzed for IL-6 and TNF cytokine content using commercially available Duoset ELISA kits (R&D Systems) according to the manufacturer’s instructions. The Quantikine Human LAP (TGF-β1) Immunoassay (R&D systems) was used to measure total TGF-β1 in acid activated cell culture supernatants. IL-10 cytokine content was analyzed using the commercially available Kit IL-10 Ready Set-Go (Ebioscience) according to the manufacturer’s instructions. The results are expressed as an average of triplicate wells ± standard error of the mean (SEM).

#### Reverse-transcription PCR

Highly purified CD19^+^ B cells (<90% purities) were left unstimulated or stimulated in the presence of CpG-B 2006 (10 μg/mL) + anti-Ig (10 μg/mL) in 24-well flat-bottom plates for 3 h at 37 °C. Total RNA was extracted with TRIzol reagent (Invitrogen), or using ReliaPrep RNA Cell Miniprep System (Promega) according to the manufacturer’s instructions, and reverse-transcribed into cDNA using iScript™ cDNA Synthesis Kit (Bio-Rad Laboratories). Real-time PCRs were performed using Power SYBR Green PCR Master Mix Reagent (Applied Biosystems) on a 7500 Real-time PCR system (Applied Biosystems). The human TGF-β1 forward and reverse primers were 5'-CCCAGCATCTGCAAAGCTC-3' and 5'-GTCAATGTACAGCTGCCGCA-3', alongside primers targeting the internal normalizer gene β-actin (Hs_ACTB_2_SG Quantitect Primer Assay, QT01680476; Qiagen). Relative expression levels were calculated for each gene using the ΔΔ^Ct^ method.

#### Statistical analysis

Data are presented as mean ± SEM. Comparisons between sample means were performed by Student’s *t* test. Values of *p* < 0.05 were considered statistically significant.

## Results

### Induction of IL-10 production by human blood memory B cells following CpG-B + anti-Ig in vitro stimulation

**Fig. 1:**
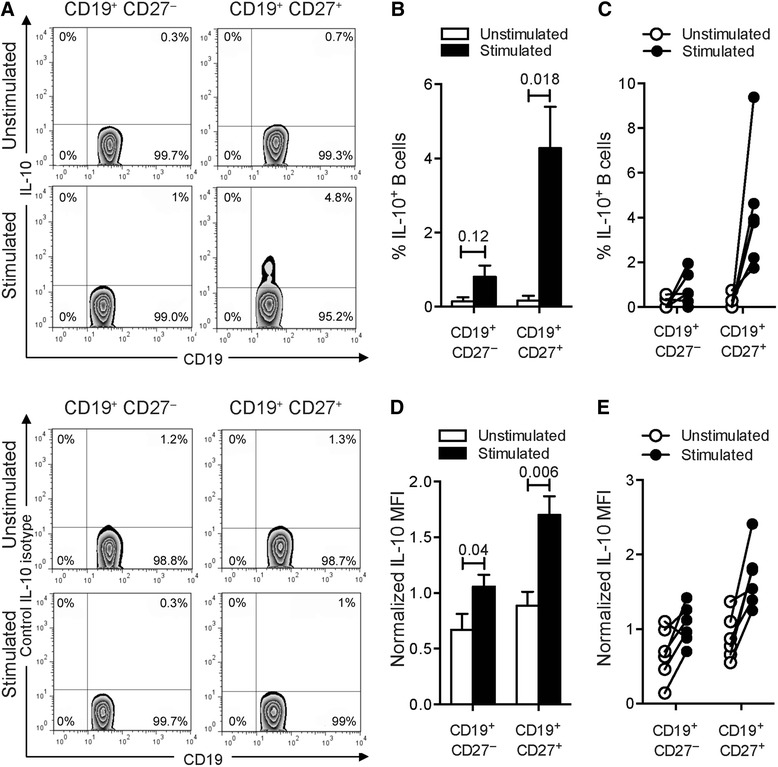
IL-10–producing B cells from human blood. **a** Frequencies of IL-10-producing B cells from human blood. Cells were cultured with medium or with combined CpG-B + anti-Ig for 24 h. PMA/ionomycin and monensin were added 5 h before the end of the culture. Cells were stained for surface CD19 and CD27 and intracellular IL-10 (*top panels*) or matched isotype control (*bottom panels*). The representative dot plots show frequencies of IL-10-producing cells among CD19^+^CD27^−^ or CD19^+^CD27^+^ B cells. **b** The *bar graph* indicates mean (± SEM) percentages of B cells that labeled positively for IL-10 (*n* = 6). Percentages presented are corrected for isotype control staining. **c** Line plot of frequencies of IL-10-producing cells among CD19^+^CD27^−^ or CD19^+^CD27^+^ B cells. Paired data are shown interconnected. **d** The *bar graph* indicates quantification (mean ± SEM) of IL-10 staining in both CD19^+^CD27^−^ and CD19^+^CD27^+^ B cells (*n* = 6). Geometric mean fluorescence intensities (MFI) were normalized to the isotype control. **e** The *line plot* shows normalized IL-10 MFI by cells among CD19^+^CD27^−^ and CD19^+^CD27^+^ B cells. Paired data are shown interconnected. Significant differences using Student’s *t* test between sample means are indicated

We assessed the ability of purified human blood B cells to produce IL-10 under both basal culture conditions and after stimulation with a combination of CpG-B, a ligand for human B cell TLR9, and anti-Ig antibodies. In the basal unstimulated state, IL-10–producing B cells were barely detected (Fig. 1a–c and Table 1). After stimulation, low but detectable frequencies of B cells expressing IL-10 were found (Fig. 1a–c and Table 1). However, the combination of CpG-B and anti-Ig did not statistically increase the percentages of CD19^+^CD27^−^ B cells expressing IL-10 (Fig. 1a–c and Table 1). As previously reported [51], the frequency of IL-10^+^ B cells was four to six times higher in the CD19^+^CD27^+^ subpopulation than in the CD19^+^CD27^−^ subset (Table 1). The frequency of IL-10–producing CD19^+^CD27^+^ B cells was comparable to that previously reported [51]. Moreover, we noted that stimulating circulating B cells with the combination of activators statistically increased within CD19^+^CD27^+^ B cells the amount of IL-10 produced per cell (Fig. 1d, e and Table 2). While a similar pattern was observed for CD19^+^CD27^−^ B cells expressing IL-10, the increased levels following B cell stimulation were not as important (Fig. 1d).

**Table 1:** Decreased frequencies of TGF-β1-expressing B cells upon in vitro polyclonal stimulation

	% IL-10		% TGF-β1		% LAP (TGF-β1)	
	CD19^+^ CD27^−^	CD19^+^ CD27^+^	CD19^+^ CD27^−^	CD19^+^ CD27^+^	CD19^+^ CD27^−^	CD19^+^ CD27^+^
Unstimulated	0.15 ± 0.09	0.17 ± 0.11	3.54 ± 0.69	4.15 ± 0.56	5.43 ± 1.11	5.40 ± 1.37
	[0.00–0.56]	[0.00–0.74]	[1.80–7.07]	[2.82–6.93]	[1.74–9.92]	[1.58–11.12]
Stimulated	0.81 ± 0.28	4.28 ± 1.02	1.20 ± 0.12	1.86 ± 0.40	3.74 ± 1.19	3.86 ± 1.18
	[0.00–1.95]	[1.75–9.38]	[0.82–1.57]	[0.82–3.28]	[0.84–9.05]	[1.03–8.53]

Values indicate frequencies (mean ± SEM) with range (number in parentheses) of cytokine-producing B cells in both CD19^+^CD27^−^ and CD19^+^CD27^+^ B cell subpopulations (*n* = 6). Frequencies were normalized to the isotype control

**Table 2:** Activation of human B cells restrains TGF-β1 production

	Normalized IL-10 MFI		Normalized TGF-β1 MFI		Normalized LAP (TGF-β1) MFI	
	CD19^+^ CD27^−^	CD19^+^ CD27^+^	CD19^+^ CD27^−^	CD19^+^ CD27^+^	CD19^+^ CD27^−^	CD19^+^ CD27^+^
Unstimulated	0.67 ± 0.13	0.89 ± 0.11	0.30 ± 0.02	0.34 ± 0.02	3.06 ± 0.36	2.59 ± 0.40
	[0.14–1.10]	[0.55–1.37]	[0.25–0.38]	[0.29–0.44]	[2.18–4.75]	[1.63–4.32]
Stimulated	1.06 ± 0.10	1.70 ± 0.15	0.15 ± 0.01	0.18 ± 0.02	2.38 ± 0.22	2.27 ± 0.31
	[0.70–1.42]	[1.25–2.41]	[0.11–0.17]	[0.13–0.22]	[1.78–3.35]	[1.52–3.68]

Values indicate quantification (mean ± SEM) with range (number in parentheses) of cytokine staining in both CD19^+^CD27^−^ and CD19^+^CD27^+^ B cells (*n* = 6). Geometric mean fluorescence intensities (MFI) were normalized to the isotype control

### Increased IL-6 and TNF production in human B cells stimulated with CpG-B and anti-Ig

**Fig. 2:**
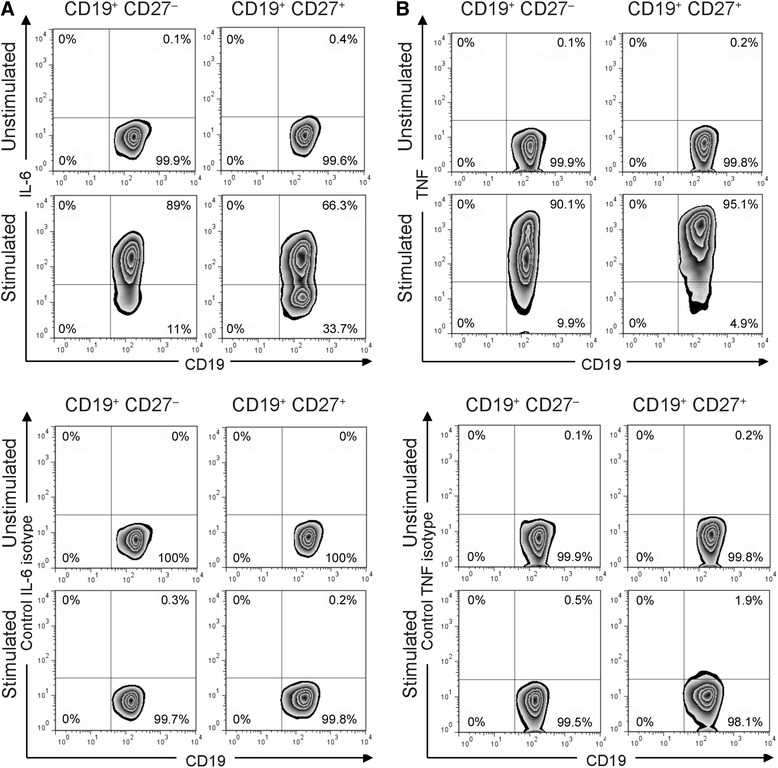
Combined CpG-B + anti-Ig stimulation potently trigger IL-6 and TNF production by B cells in vitro. Frequencies of (**a**) IL-6- and (**b**) TNF-producing B cells from human blood. Cells were cultured with medium or with combined CpG-B + anti-Ig for 24 h. PMA/ionomycin and monensin were added 5 h before the end of the culture. Cells were stained for surface CD19 and CD27 and intracellular IL-6 or TNF (*top panels*) or matched isotype control (*bottom panels*). The representative dot plots show frequencies of IL-6- and TNF-producing cells among CD19^+^CD27^−^ or CD19^+^CD27^+^ B cells

We next evaluated the capacity of purified circulating B cells to produce the proinflammatory cytokines IL-6 and TNF under basal and stimulated conditions. IL-6 production by B cells is a major pathogenicity factor for B cells in CNS autoimmunity [3, 5]. TNF is also considered as an important factor that is secreted by active B cells in MS [13]. Analysis of frequencies of B cells under resting conditions revealed that B cells did not produce IL-6 (Fig. 2a) or TNF (Fig. 2b). Coordinated stimulation of BCR and TLR9 however drastically increased IL-6 and TNF levels in these cells. In contrast to IL-10 production, which was confined to stimulated CD19^+^CD27^+^ B cells, high expression levels of IL-6 and TNF were found in both CD19^+^CD27^+^ and CD19^+^CD27^−^ subsets. These data suggest that polyclonal B cell stimulation shifts B cells towards a proinflammatory dominant phenotype. In line with these results, B cells from patients with MS were on average shown to produce more proinflammatory TNF and IL-6 and less regulatory IL-10 [5, 17].

### BCR ligation together with TLR9 engagement inhibits TGF-β1 production by human blood B cells

**Fig. 3:**
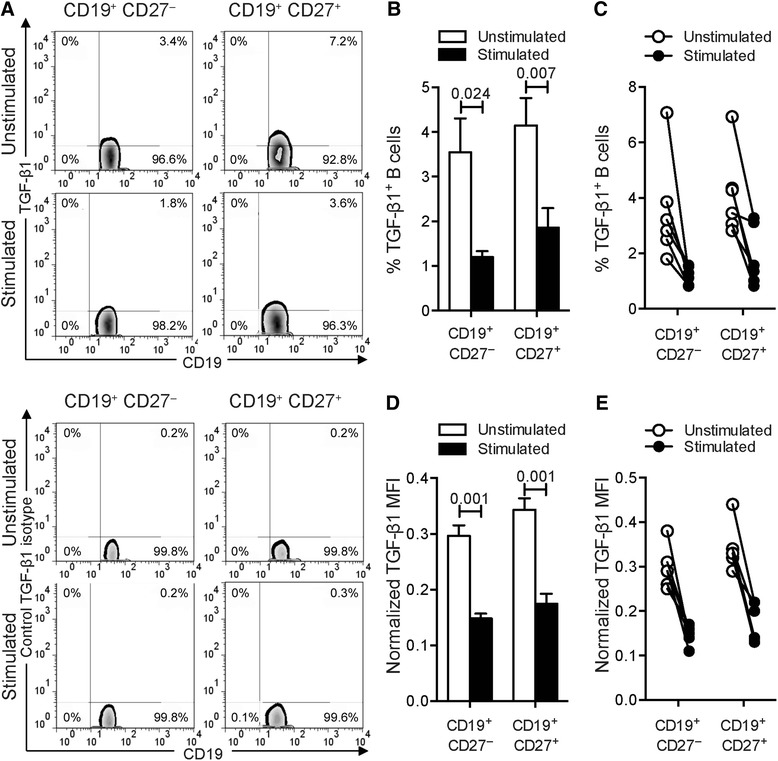
Activation of human B cells negatively regulates TGF-β1 production. **a** Frequencies of TGF-β1-producing B cells from human blood. Cells were cultured with medium or with combined CpG-B + anti-Ig for 24 h. PMA/ionomycin and monensin were added 5 h before the end of the culture. Cells were stained for surface CD19 and CD27 and intracellular TGF-β1 (*top panels*) or matched isotype control (bottom *panels*). The representative dot plots show frequencies of TGF-β1-producing cells among CD19^+^CD27^−^ or CD19^+^CD27^+^ B cells. **b** The *bar graph* indicates mean (± SEM) percentages of B cells that labeled positively for TGF-β1 (*n* = 6). Percentages presented are corrected for isotype control staining. **c** *Line plot* of frequencies of TGF-β1-producing cells among CD19^+^CD27^−^ or CD19^+^CD27^+^ B cells. *Paired data* are shown interconnected. **d** The *bar graph* indicates quantification (mean ± SEM) of TGF-β1 staining in both CD19^+^CD27^−^ and CD19^+^CD27^+^ B cells (*n* = 6). Geometric MFI were normalized to the isotype control. **e** The *line plot* shows normalized TGF-β1 MFI by cells among CD19^+^CD27^−^ and CD19^+^CD27^+^ B cells. *Paired data* are shown interconnected. Significant differences using Student’s *t* test between sample means are indicated

We next assessed the ability of human blood B cells to express TGF-β1 under our experimental conditions. In the basal unstimulated state we could readily detect TGF-β1–producing B cells (Fig. 3a–c and Table 1). The frequencies of TGF-β1^+^ B cells were comparable between the CD19^+^CD27^+^ and CD19^+^CD27^−^ subpopulations. Strikingly, TGF-β1-producing B cell proportions significantly decreased after the combined CpG and anti-Ig stimulation (Fig. 3a–c and Table 1). A comparable degree of reduction in TGF-β1 expression was observed in both naïve and memory B cells after stimulation. Furthermore, we noted that stimulating circulating B cells with the combination of activators statistically reduced the expression levels of TGF-β1 per cell, measured as mean florescence intensity (MFI) (Fig. 3d, e and Table 2). Taken together these data indicate that dual BCR ligation and TLR9 engagement induces substantial decreases in both the frequency of B cells that express TGF-β1 and the absolute levels of TGF-β1 expression by B cells.

### Analysis of cytokines secreted by human blood B cells after CpG-B and anti-Ig stimulation

**Fig. 4:**
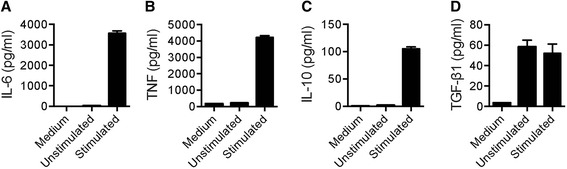
Stimulation of B cells elicits secretion of IL-6, TNF, and IL-10, but not TGF-β1. Purified B cells from human blood were cultured with serum-free medium alone or with combined CpG-B + anti-Ig for 24 h. The amount of (**a**) IL-6, (**b**) TNF, (**c**) IL-10, and (**d**) total TGF-β1 protein in the culture cell-free supernatants was determined by ELISA. *Bar graphs* show mean cytokine concentrations (± SEM) from technical triplicates from one representative donor out of two analyzed

We next measured the effect of combined CpG and anti-Ig stimulation on pro- and anti-inflammatory cytokine secretion by purified B cells. As expected, high levels of IL-6 (Fig. 4a) and TNF (Fig. 4b) were detected in supernatants from B cells cultures following stimulation. Likewise, stimulation of B cells also increased IL-10 secretion, although to a much lesser extent (Fig. 4c). As TGF-β1 is secreted in a latent form, linked to Latency Associated Protein (LAP) [52], latent TGF-β1 was analyzed by enzyme-linked immunosorbent assay (ELISA) after dissociation of TGF-β1 from LAP by acidification of supernatant samples. This method measures total TGF-β1, equivalent to dissociated latent TGF-β1 plus any free TGF-β1 present prior to acidification. Compared to control serum-free conditions, low concentrations of total TGF-β1 were detected in cell-free supernatants of resting B cells (Fig. 4d). Under these conditions, levels of total TGF-β1 secreted by resting B cells was not inferior to those measured by stimulated B cells (Fig. 4d).

### Reduced TGF-β1 expression in human circulating B cells following activation

**Fig. 5:**
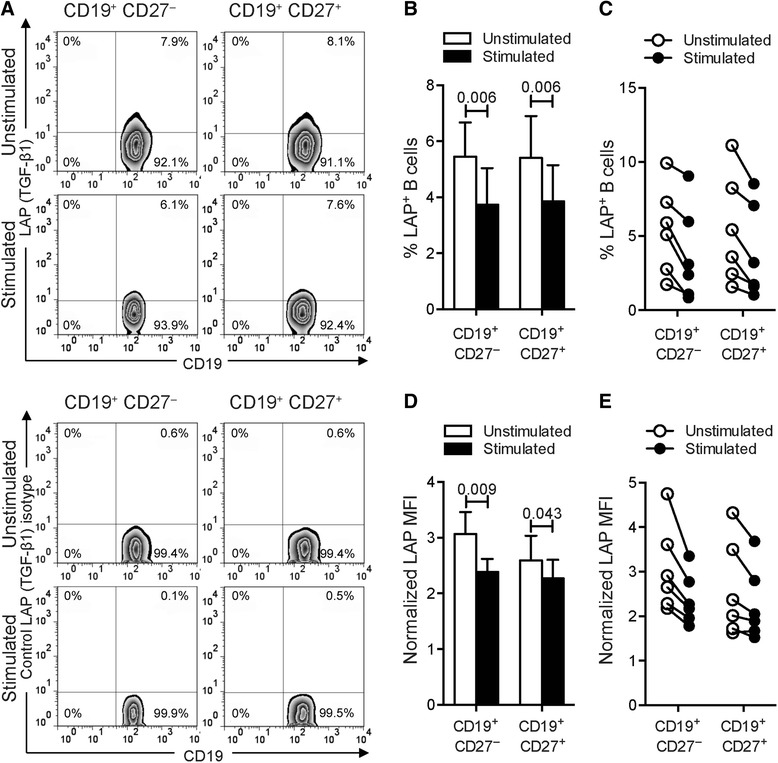
Stimulation of B cells restrains LAP expression. **a** Frequencies of LAP-producing B cells from human blood. Cells were cultured with medium or with combined CpG-B + anti-Ig for 24 h. PMA/ionomycin was added 5 h before the end of the culture. Cells were stained for surface CD19, CD27, and LAP (*top panels*) or matched isotype control (*bottom panels*). The representative dot plots show frequencies of LAP–expressing cells among CD19^+^CD27^−^ or CD19^+^CD27^+^ B cells. **b** The *bar graph* indicates mean (± SEM) percentages of B cells that labeled positively for LAP (*n* = 6). Percentages presented are corrected for isotype control staining. **c** *Line plot* of frequencies of LAP-expressing cells among CD19^+^CD27^−^ or CD19^+^CD27^+^ B cells. *Paired data* are shown interconnected. **d** The *bar graph* indicates quantification (mean ± SEM) of LAP staining in both CD19^+^CD27^−^ and CD19^+^CD27^+^ B cells (*n* = 6). Geometric MFI were normalized to the isotype control. **e** *Line plot* shows normalized LAP MFI by cells among CD19^+^CD27^−^ and CD19^+^CD27^+^ B cells. *Paired data* are shown interconnected. Significant differences using Student’s *t* test between sample means are indicated

As TGF-β1 is produced in a latent form, linked LAP, and is predominantly expressed on the surface of TGF-β1-producing cells [52], we next evaluated the cell-surface expression of LAP–TGF-β1 on B cells by flow cytometry. Comparable frequencies of LAP–TGF-β1^+^ B cells were seen in unstimulated CD19^+^CD27^+^ and CD19^+^CD27^−^ subpopulations (Fig. 5a and Table 1). Remarkably, B cell stimulation significantly reduced the percentage of CD19^+^CD27^+^ and CD19^+^CD27^−^ B cells bearing LAP–TGF-β1 (Fig. 5a–c and Table 1). Moreover, we observed a substantial decreased density (MFI) of cell-surface expression of LAP–TGF-β1 on naïve CD19^+^CD27^−^ B cells (Fig. 5d, e and Table 2), which were significantly more abundant in peripheral blood than memory B cells (Additional file 1: Figures S1A-B). A similar trend, with poorer correlation, was observed for expression levels of LAP–TGF-β1 per cell within the CD19^+^CD27^+^ B cell subpopulation (Fig. 5d, e and Table 2). Consistent with these data, B cell stimulation significantly reduced the level of TGF-β1 messenger RNA (mRNA) by whole CD19^+^ B cells as measured by quantitative reverse transcription real-time polymerase chain reaction (PCR) (Additional file 1: Figure S1C). Altogether, our results indicate that activation of human circulating B cells through BCR/TLR9 co-engagement, a remarkably potent mechanism of activation of autoreactive B cells [53], shifts B cells from a regulatory/suppressive phenotype associated with TGF-β1 expression to a proinflammatory state characterized by low expression levels of TGF-β1.

## Discussion

Data indicate that depending on the signals B cells receive, proinflammatory or immunoregulatory cytokines can be produced, and the shift towards an inflammatory or a protective/suppressive immune response will occur [54]. The goal of this investigation was to evaluate the ability of human blood B cells to produce the classical pro- and anti-inflammatory cytokines IL-6, TNF, IL-10, and TGF-β1, respectively, under both basal and stimulated conditions. We found that a combination of adaptive signals by BCR cross-linking and innate signals through TLR9 engagement strongly induced IL-6 and TNF production by human B cells in vitro. Although modest, the signals induced by CpG-DNA and BCR ligation also caused induction of IL-10 production by memory B cells. Strikingly, stimulation of B cells by engagement of BCR and TLR9 decreased TGF-β1 production. In regards to the implication of TGF-β1 expression in regulatory B cell functions in immune tolerance [36, 38, 55, 56], the present findings imply that loss of B cell-derived TGF-β1 production following B cell activation may confer increased susceptibility to autoimmune responses.

Accumulating evidence from both mouse and human studies confirms the existence of regulatory B cells, and is beginning to define their mechanisms of action. In EAE, a CD4^+^ T cell-mediated autoimmune disease and a well-established model of MS, data indicate that separate B cell subsets regulate the induction and recovery of autoimmune neuroinflammation [35, 57–61]. While initially all regulatory functions of suppresive B cells were attributed to their capabilities to produce IL-10, recent studies have highlighted additional mechanisms by which Bregs suppress immunity. In addition to the recently reported regulatory role of IL-35-producing B cells during disease recovery [61], data from our group have established that TGF-β1-producing B cells restrain the initiation phase of EAE and the development of Th17 cells [36]. Consistent with a regulatory role of B cell-derived of TGF-β1 production in CNS autoimmunity, B cells were previously found to have a central function in induction of oral tolerance and amelioration of EAE via the up-regulation of TGF-β in gut associated lymphoid tissue (GALT) [44, 62]. Moreover, other related work has reported that TGF-β1 negatively regulates the encephalitogenic capacity of Th17 cells [63] and EAE [64–66].

Although data indicate that TGF-β1-producing B cells exert a regulatory function in EAE, their relevance to MS is yet to be determined. Due to its prominent role in controlling the immune system [67], TGF-β1 has been suggested to be one of the central regulatory cytokines in MS. Interestingly, data obtained from MS patients have reported that TGF-β expression is increased in peripheral blood mononuclear cells (PBMCs) from MS patients with slight or no disability [68] or stable disease [69]. In a longitudinal study, TGF-β expression was further shown to decline in relapse and to return to baseline values four to eight weeks following the MS relapse [70]. Of note, myelin-stimulated peripheral blood T cell clones from MS patients failed to secrete TGF-β during acute attacks, while TGF-β expression was restored during disease remission [71]. While no data to date have investigated TGF-β production by B cells in MS, our findings, despite being derived from healthy people, indicate that activation of human blood B cells reduced their capacity to produce TGF-β1. Considering the importance of TGF-β1 in modulating immunity and the abnormal proinflammatory polarization of B cells of MS patients [5, 6, 13, 17], inefficient production of TGF-β1 by B cells may be associated with an increased risk of developing MS or driving new relapsing MS disease activity. It is also tempting to postulate that exacerbation of symptoms, or onset of new pathologies following B cell depletion with Rituximab in a few patients with immune-mediated diseases [26–29], could be associated with the depletion of unactivated TGF-β1-producing B cells. Future studies aimed at evaluating TGF-β1 production by B cells, regardless of their antigenic specificity, from MS subjects and healthy controls in response to modes of activation relevant to B-cell–T-cell interaction and MS [13, 17, 72] will allow for a better understanding of the contribution of B cell-derived TGF-β1 in this disease.

The phenotypic characterization of human IL-10-producing B cells has been integral in accelerating assessment of their function and relevance in immune-mediated diseases [73]. B cells isolated according to the cell surface expression of receptors classically used to separate known B cell subsets have revealed that CD19^+^CD27^−^ naïve-like B cells produced more IL-10 than CD19^+^CD27^+^ B cells after stimulation via BCR and CD40 [17]. In a separate study, it was shown that the proportion of IL-10-producing B cells was higher in the CD19^+^CD27^+^ subpopulation than in the CD19^+^CD27^−^ subset after stimulation with CpG-DNA and BCR ligation [51]. These different results may stem from the use of distinct stimulatory conditions. Here, our data confirm that IL-10-producing B cells activated via BCR and TLR9 fall within the CD19^+^CD27^+^ subpopulation [51]. Using CD27 as a surrogate marker of human memory B cells, our data demonstrate that TGF-β1-producing B cells do not belong to a unique B cell subset. The characterization of human TGF-β1-producing B cells may provide a better understanding of their function and development. Interestingly, the quantification of TGF-β1 expression by B cells in EAE mice [36] indicate that murine TGF-β1-producing B cells do not belong to the CD1d^hi^CD5^+^ B10 subset [74] or CD138^+^ plasma cells, which have been shown to be the main source of IL-10 and IL-35 during disease development [61, 75].

## Conclusions

In summary, our data demonstrate that B cell activation by BCR/TLR9 co-engagement differentially regulates the production of pro- and anti-inflammatory cytokines by human B cells. B cell stimulation with combined CpG-B + anti-Ig induced strong IL-6 and TNF production. IL-10 was modestly elevated after B cell activation. Remarkably, B cells stimulated in this way had a significantly reduced capacity to produce TGF-β1. These findings imply that activation of human B cells may amplify autoimmune responses by restricting the regulatory functions of resting TGF-β1-producing B cells and favoring the proinflammatory activities of B cells. These observations further support the consensus that the action of naïve B cells is fundamentally regulatory and that activated B cells preferentially produce proinflammatory cytokines [6, 17].

## Additional files

Additional file 1: Figure S1. B cell activation reduced TGF-β1 mRNA expression. **a** A representative gating of naïve and memory B cells in PBMCs is shown. Cells were first gated for singlets (FSC-H vs. FSC-A) and lymphocytes (SSC-A vs. FSC-A), followed by a live/dead gate. Naïve B cells (CD27^–^) and resting and activated memory B cells (CD27^+^) were distinguished by CD19 versus CD27 gating. FSC-H, forward scatter height; FSC-A, forward scatter area; SSC-A, side scatter area. **b** Scatter plot indicates mean (± SEM) percentages of naïve (CD27^–^) versus memory (CD27^+^) B cell subpopulations (*n* = 6). **c** Quantitative RT-PCR analysis of human B cells for the expression of TGF-β1. Expression levels are normalized using the ΔΔ^Ct^ method, relative to β-actin. Results show mean of 6 independent sets of samples. Errors bars represent SEM. (DOCX 286 kb)

## Abbreviations

Ab: Antibodies
APRIL: A proliferation-inducing ligand
BAFF: B-cellactivating factor
BCDT: B cell-depleting therapies
BCR: B cell receptor
CNS: Central nervous system
EAE: Experimental autoimmune encephalomyelitis
ELISA: Enzyme-linked immunosorbent assay
GM-CSF: Granulocyte-macrophage colony-stimulating factor
IL: Interleukin
LAP: Latency associated protein
MFI: Mean florescence intensity
MS: Multiple sclerosis
PCR: Polymerase chain reaction
PMA: Phorbol 12-myristate 13-acetate
TGF: Transforming growth factor
TLR: Toll-like receptor
TNF: Tumor necrosis factor
