# Supplementary material for: Activation of human B cells negatively regulates TGF-β1 production
Source: J Neuroinflammation. 2017 Jan 19;14:13. doi: 10.1186/s12974-017-0798-5 (PMC5244520; doi:10.1186/s12974-017-0798-5)
Supplement: Additional file 1: Figure S1. — B cell activation reduced TGF-β1 mRNA expression. a A representative gating of naïve and memory B cells in PBMCs is shown. Cells were first gated for singlets (FSC-H vs. FSC-A) and lymphocytes (SSC-A vs. FSC-A), followed by a live/dead gate. Naïve B cells (CD27–) and resting and activated memory B cells (CD27+) were distinguished by CD19 versus CD27 gating. FSC-H, forward scatter height; FSC-A, forward scatter area; SSC-A, side scatter area. b Scatter plot indicates mean (± SEM) percentages of naïve (CD27–) versus memory (CD27+) B cell subpopulations (n = 6). c Quantitative RT-PCR analysis of human B cells for the expression of TGF-β1. Expression levels are normalized using the ΔΔCt method, relative to β-actin. Results show mean of 6 independent sets of samples. Errors bars represent SEM. (DOCX 286 kb) [file 12974_2017_798_MOESM1_ESM.docx]

**Supplementary Material**

**
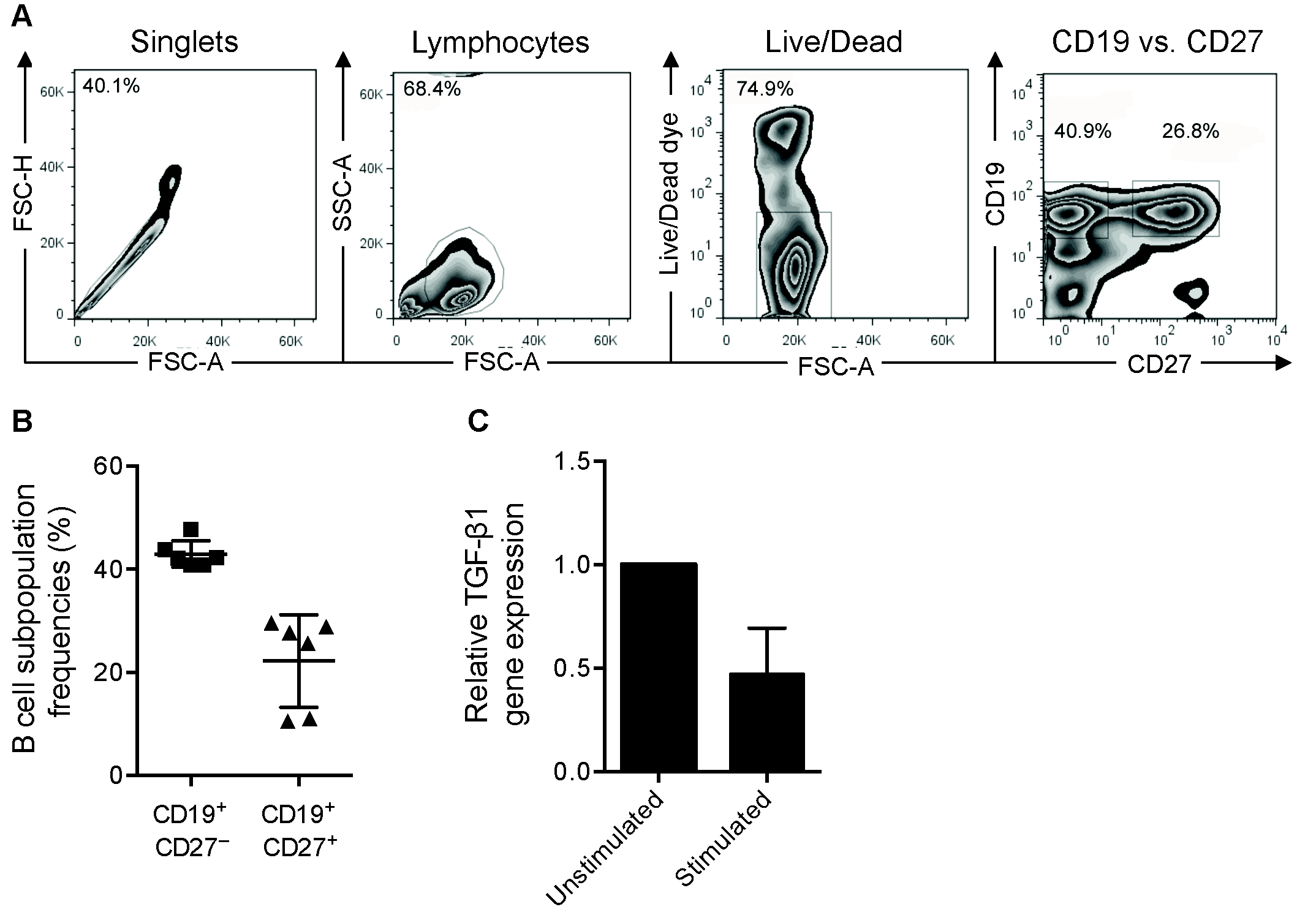
Supplementary Figure S1: B cell activation reduced TGF-β1 mRNA expression.** (**A**) A representative gating of naïve and memory B cells in PBMCs is shown. Cells were first gated for singlets (FSC-H vs. FSC-A) and lymphocytes (SSC-A vs. FSC-A), followed by a live/dead gate. Naïve B cells (CD27^–^) and resting and activated memory B cells (CD27^+^) were distinguished by CD19 versus CD27 gating. FSC-H, forward scatter height; FSC-A, forward scatter area; SSC-A, side scatter area. (**B**) Scatter plot indicates mean (± SEM) percentages of naïve (CD27^–^) versus memory (CD27^+^) B cell subpopulations (n = 6). (**C**) Quantitative RT-PCR analysis of human B cells for the expression of TGF-β1. Expression levels are normalized using the ΔΔ^Ct^ method, relative to β-actin. Results show mean of 6 independent sets of samples. Errors bars represent SEM.
